# The prognostic value of sarcopenia combined with preoperative fibrinogen–albumin ratio in patients with intrahepatic cholangiocarcinoma after surgery: A multicenter, prospective study

**DOI:** 10.1002/cam4.4035

**Published:** 2021-06-08

**Authors:** Haitao Yu, Mingxun Wang, Yi Wang, Jinhuan Yang, Liming Deng, Wenming Bao, Bangjie He, Zixia Lin, Ziyan Chen, Kaiyu Chen, Baofu Zhang, Fangting Liu, Zhengping Yu, Longyun Ye, Bin Jin, Gang Chen

**Affiliations:** ^1^ Department of Hepatobiliary Surgery The First Affiliated Hospital of Wenzhou Medical University Wenzhou China; ^2^ Key Laboratory of Diagnosis and Treatment of Severe Hepato‐Pancreatic Diseases of Zhejiang Province The First Affiliated Hospital of Wenzhou Medical University Wenzhou China; ^3^ Department of Epidemiology and Biostatistics, School of Public Health and Management Wenzhou Medical University Wenzhou China; ^4^ Department of Liver Transplantation Qilu Hospital of Shandong University Jinan China

**Keywords:** fibrinogen–albumin ratio (FAR), intrahepatic cholangiocarcinoma, nomogram, prognosis, sarcopenia

## Abstract

**Background:**

To explore the prognostic value of the fibrinogen–albumin ratio (FAR) combined with sarcopenia in intrahepatic cholangiocarcinoma (ICC) patients after surgery and to develop a nomogram for predicting the survival of ICC patients.

**Materials and Methods:**

In this prospective cohort study, 116 ICC patients who underwent radical surgery were enrolled as the discovery cohort and another independent cohort of 68 ICC patients was used as the validation cohort. Kaplan–Meier method was used to analyze prognosis. The independent predictor of overall survival (OS) and recurrence‐free survival (RFS) was evaluated by univariable and multivariable Cox regression analyses, then developing nomograms. The performance of nomograms was evaluated by concordance index (C‐index), calibration curve, receiver operating characteristic curve analysis (ROC), and decision curve analysis (DCA).

**Results:**

Patients with high FAR had lower OS and RFS. FAR and sarcopenia were effective predictors of OS and RFS. Patients with high FAR and sarcopenia had a poorer prognosis than other patients. OS nomogram was constructed based on age, FAR, and sarcopenia. RFS nomogram was constructed based on FAR and sarcopenia. C‐index for the nomograms of OS and RFS was 0.713 and 0.686. Calibration curves revealed great consistency between actual survival and nomogram prediction. The area under ROC curve (AUC) for the nomograms of OS and RFS was 0.796 and 0.791 in the discovery cohort, 0.823 and 0.726 in the validation cohort. The clinical value of nomograms was confirmed by the DCA.

**Conclusions:**

ICC patients with high FAR and sarcopenia had a poor prognosis, the nomograms developed based on these two factors were accurate and clinically useful in ICC patients who underwent radical resection.

## Graphical abstract

Sarcopenia and high levels of FAR are related to the poor prognosis of ICC patients undergoing radical surgery. FAR and sarcopenia are convenient, inexpensive, and reliable marks that provide references for improving the prognosis and new treatment strategies for ICC patients after surgery.

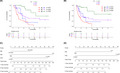

## 1 INTRODUCTION

Intrahepatic cholangiocarcinoma (ICC) is the second most common primary malignant tumor in the liver after hepatocellular carcinoma.^1^ In the past two decades, the incidence of ICC has risen worldwide,^2^ however, the prognosis of ICC patients is worse than other cancers, and the 5‐year survival rate after diagnosis is below 10%.^3^ Radical surgery and liver transplantation are the only effective treatments for ICC, but only about 35% of patients are eligible for these treatments.^3^, ^4^ At present, the 5‐year overall survival of ICC patients after radical resection is 25%–40%.^5^ The factors affecting the prognosis of ICC patients who accepted radical surgery are still unclear, hence, it is particularly significant to find non‐invasive and convenient factors that affect the prognosis of patients with ICC, and according to these indicators to establish an effective treatment strategy after surgery.

The survival time of patients with malignant tumor is associated with many factors, such as systemic inflammation indicators and nutritional status. For cancer patients, malnutrition is a serious problem, it will directly lead to cachexia, worse treatment effect, and shorter survival time.^6^ At the same time, circulating inflammatory markers that mediate systemic inflammatory response play a vital role in the occurrence and development of many cancers. They destroy human immunity, reduce tumor response to cytotoxic drugs, and ultimately shorten the survival time of patients with ICC.^7^, ^8^, ^9^, ^10^ Albumin and fibrinogen are the two most common biomarkers of inflammation. Recent studies reported that albumin was associated with the prognosis of many diseases, such as Oral Cavity cancer,^11^ metastatic pathological femur fractures,^12^ and amyotrophic lateral sclerosis.^13^ Besides, previous studies showed that fibrinogen has the function of predicting the prognosis of diseases, such as spontaneous cerebral hemorrhage^14^ and renal cancer.^15^ More and more studies have shown that high fibrinogen and low albumin are correlated with poor prognosis in cancer patients. As a new prognostic immune biomarker, the fibrinogen–albumin ratio (FAR) has more prognostic value than high fibrinogen or low serum albumin. In fact, FAR has been reported as a potential prognostic factor for many malignant tumors, such as gallbladder cancer,^16^ breast cancer,^17^ colorectal cancer,^18^ and pancreatic cancer.^19^ However, the relationship between FAR and the prognosis of patients with ICC has not yet been explored.

Sarcopenia is a disease characterized by a decline in body muscle mass and loss of function.^20^ The loss of muscle mass is one of the three major phenotypes of malnutrition.^21^ Recent studies suggested that sarcopenia was associated with the poor prognosis of many malignant tumors, such as esophageal cancer,^22^ colorectal cancer,^23^ and hepatocellular carcinoma.^24^ Sarcopenia and inflammation marker FAR are usually used alone as prognostic factors instead of building a combined model to predict the prognosis of cancer patients.

Recently, we had also proved the prognostic value of sarcopenia in ICC patients, and sarcopenia combined with hepatolithiasis may have better predictive potential.^25^ In this study, our purpose was to explore the relationship between the inflammatory biomarker FAR and the prognosis of ICC patients, and to explore whether sarcopenia combined with FAR can accurately predict the prognosis of ICC patients after radical resection. Moreover, we had developed and verified the nomograms based on these two factors to accurately predict the OS and RFS of ICC patients who accepted the radical surgery.

## 2 MATERIALS AND METHODS

### 2.1 Patients

A total of 234 ICC patients who underwent surgery were recruited for the discovery cohort and validation cohort during the study period in two centers. The consecutive recruitment of the discovery cohort of this study started in August 2012, after the selection of exclusion criteria, 116 patients who underwent radical surgery at the First Affiliated Hospital of Wenzhou Medical University from January 2013 to October 2019 were finally included in the discovery cohort. From August 2015 to October 2019, an independent validation cohort of 68 patients was screened from Qilu Hospital of Shandong University using the same criteria as that for the discovery cohort. The inclusion criteria of ICC patients were as follows: (a) histologically confirmed as primary intrahepatic cholangiocarcinoma with R0 radical resection; (b) no anti‐tumor therapy such as chemotherapy and radiotherapy before radical resection; (c) no uncontrollable acute infection, chronic renal failure, hematopoietic system disease or other malignant tumors; (d) complete clinical data available at the time of the first diagnosis, including hematological indicators and plain CT image to analyze psoas muscle index (PMI). The exclusion criteria of ICC patients in this study were: (a) pathologically confirmed not to be primary ICC or combined with hepatocellular carcinoma; (b) perioperative death; (c) underwent palliative surgery or R1/R2 resection; (d) received preoperative antitumor therapy; (e) loss of follow‐up; (f) missing value in clinical data including lymph node metastasis, CT image data, and hematological indicators.

### 2.2 Preoperative anthropometric and image analysis

Physical status and laboratory indicators are obtained within 2 weeks before surgery. BMI is calculated by dividing weight (kg) by the square of height (m^2^). We use the total psoas muscle mass areas (PMS) as an index to judge the patient's muscle mass in this study.^26^, ^27^, ^28^ PMI is used as a simple indicator to define sarcopenia, the calculation formula of PMI is as follows:

$$\text{PMI}=\text{total psoas muscle mass areas}\;\left({\text{cm}}^{2}\right)/{\text{hight}}^{2}\left(m^{2}\right).$$

We analyzed the plain CT image at the level of the third lumbar vertebra to calculate the PMS, PMS was calculated as follows:

PMS=a×b×π,

where a and b are the radii of the major and minor axes, respectively.^29^ All preoperative plain CT imaging was obtained within 1 month before surgery and the CT images were analyzed by two physicians separately.

### 2.3 Data collection

The clinicopathological information of each patient is obtained from the medical records, including: age, gender, TNM stage, tumor differentiation, tumor size, tumor number, HBsAg, lymph node invasion, vascular invasion, BMI, AFP, CEA, CA19‐9, smoking history, drinking history, liver cirrhosis, perineural invasion, portal hypertension, Child‐Pugh grade, serum albumin, FAR, and PMI. TNM stage is according to the 8th edition of the American Joint Committee on Cancer (AJCC) staging system for ICC. Lymph node invasion, vascular invasion, perineural invasion, and liver cirrhosis were detected by the pathology of postoperative specimens. The discovery outcomes in this study were OS and RFS, OS was defined as the period from the day of surgery to death or the last follow‐up, RFS was defined as the period between the date of surgical resection and date of ICC relapse or death. The deadline for follow‐up was 20th May 2020.

### 2.4 Ethics statement

This study was approved by the Medical Ethics Committee of the First Affiliated Hospital of Wenzhou Medical University and Qilu Hospital of Shandong University. All patients signed written informed consent.

### 2.5 Statistical analysis

Normally distributed data were showed as mean±standard deviation and non‐normally distributed data were reported as medians and interquartile range. Categorical variables were presented in the form of frequencies and percentages. Comparisons of baseline characteristics between groups were tested by Pearson's chi‐square test or Fisher's exact test. The cut‐off values of FAR and PMI for OS and RFS were calculated by X‐tile software. The K–M method was carried out to analyze the OS and RFS of ICC patients. The predictive factors for OS and RFS were evaluated by univariate and multivariate Cox regression analyses. The clinically significant factors calculated from the multivariate analysis were integrated into nomograms to predict the prognosis of ICC patients who underwent radical surgery. The performance of nomograms was evaluated by the C‐index and Calibration plots. The DCA and ROC were used to assess the predictive accuracy and net benefit of the nomograms. Statistical analysis was performed using R, version 3.5.2 (R packages “rms,” “survival,” “survminer,” “timeROC,” “forestplot,” “stdca”). *p *< 0.05 was considered to be statistically significant.

## 3 RESULTS

### 3.1 FAR as a potential prognostic factor of ICC

**FIGURE 1:**
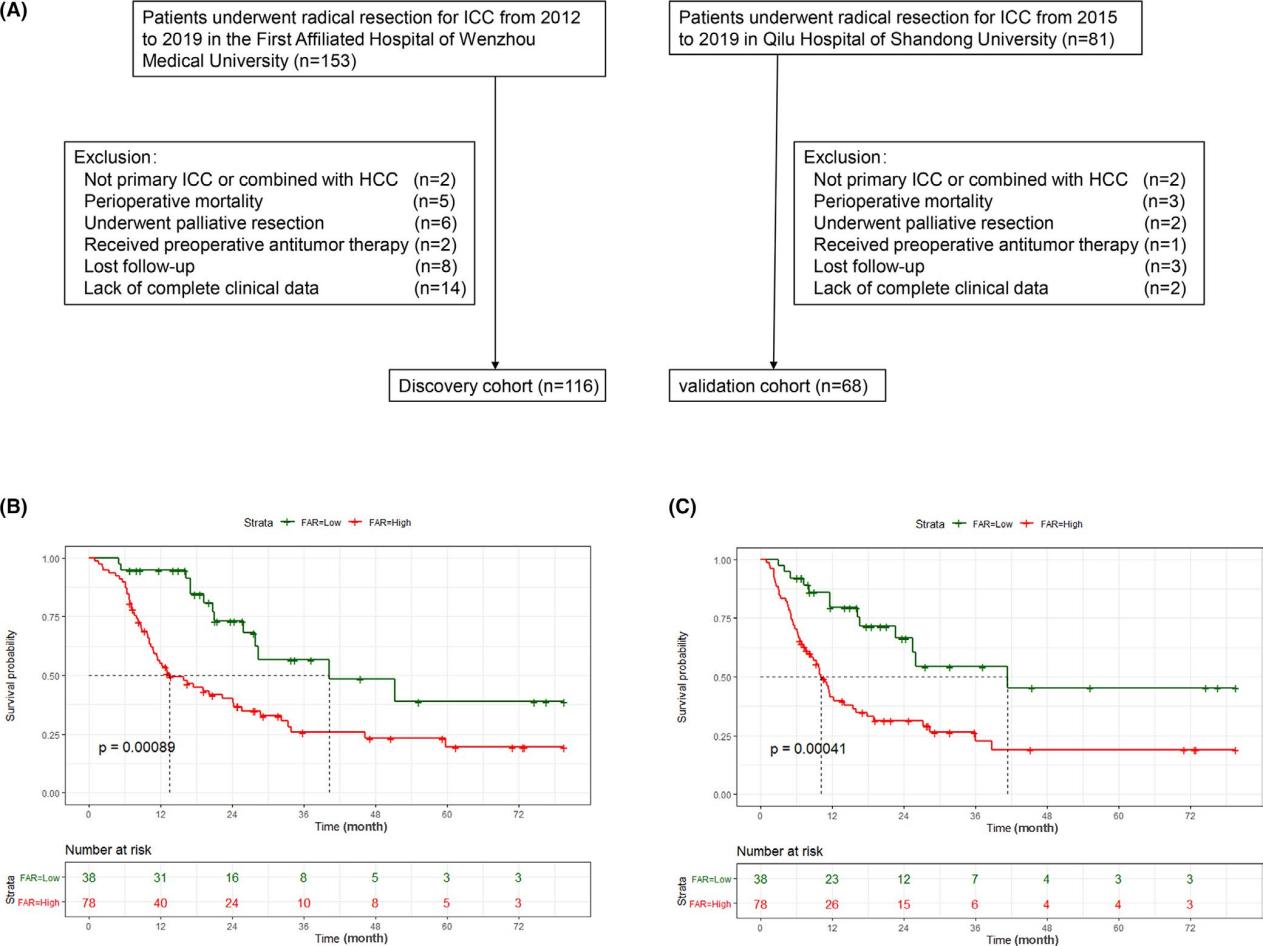
The flow diagram of the patients with discovery and validation cohorts (A). Kaplan‐Meier curves of OS (B) and RFS (C) stratified according to FAR in the discovery cohort. Abbreviations: FAR, fibrinogen‐albumin ratio; OS, overall survival; RFS, recurrence‐free survival

**FIGURE 2:**
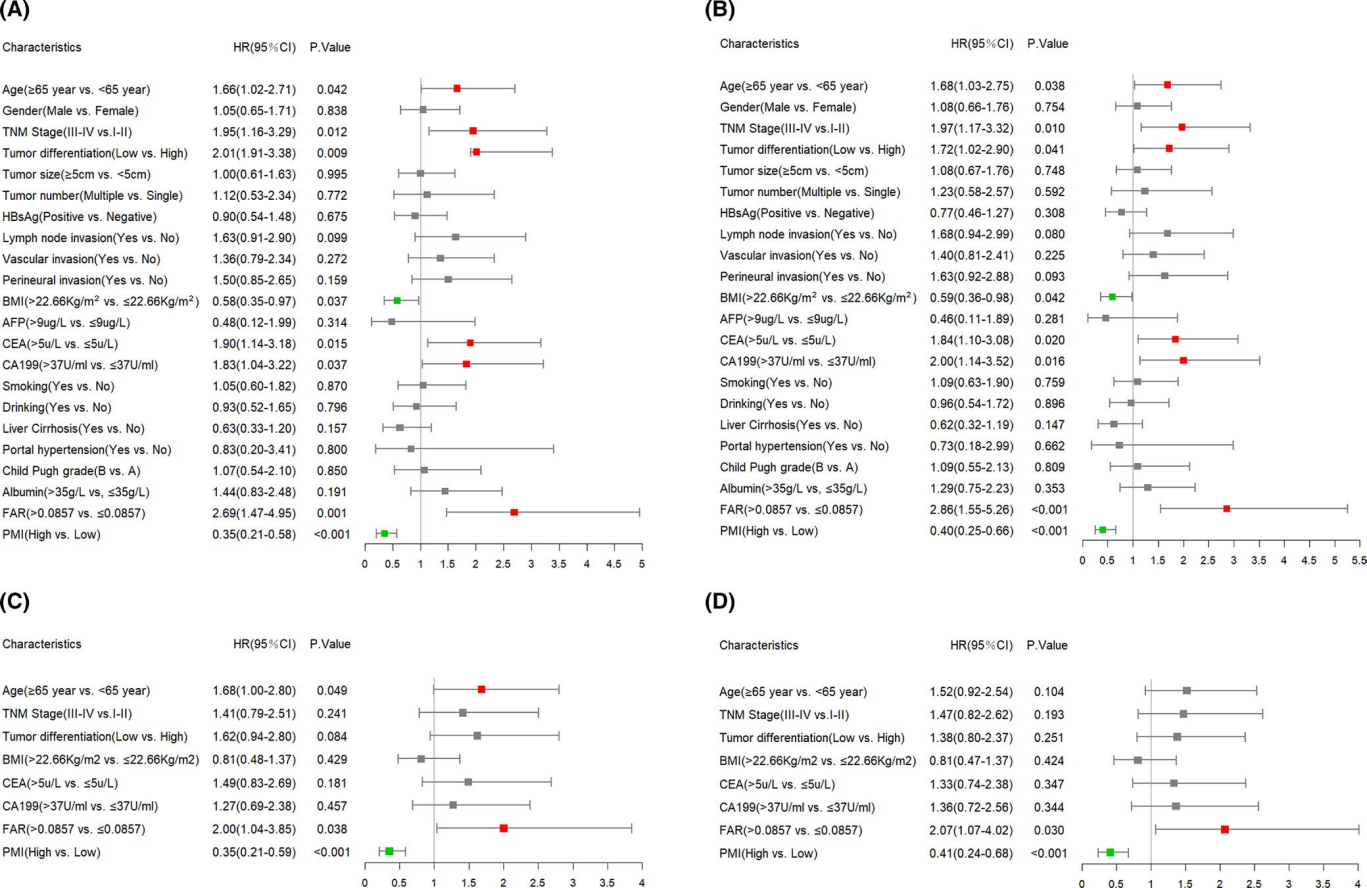
Univariate and multivariate Cox regression analysis for OS and RFS. univariate Cox regression analyses for determining the potential prognostic factors for OS (A) and RFS (B) in discovery cohort, multivariate Cox regression analyses for determining the independent potential prognostic factors for OS (C) and RFS (D) in discovery cohort. Abbreviations: AFP, alpha‐fetoprotein; BMI, body mass index; CA19‐9, Carbohydrate antigen199; CEA, carcinoembryonic antigen; FAR, fibrinogen‐albumin ratio; OS, overall survival; PMI, psoas muscle index; RFS, recurrence‐free survival

A total of 153 ICC patients who underwent surgery were recruited for the discovery cohort. After the exclusion criteria, 116 patients were finally enrolled in the discovery cohort (Figure 1A), based on the optimal cut‐off value calculated by x‐tile, the patients were divided into high‐risk group (FAR > 0.0875, *n *= 78, 67.2%) and low‐risk group (FAR≤0.0875, *n* = 38, 32.8%). The relationship between clinicopathological factors and FAR in the discovery cohort is listed in Table 1. Patients with high FAR had a poorer tumor differentiation (*p* = 0.004), higher CA19‐9 (*p* = 0.002), and lower AFP (*p* = 0.037) compared to those in the low FAR group. The K–M analysis showed that the patients in the high‐risk group had shorter OS and RFS than those in the low‐risk group (*p *< 0.001 in Figure 1B *p* < 0.001 in Figure 1C, respectively). According to univariate and multivariate Cox proportional regression analyses, FAR was an independent risk factor for OS and RFS in patients with ICC (HR: 2.00, 95% CI: 1.04–3.85, *p* = 0.038 in Figure 2C, HR: 2.07, 95% CI: 1.07–4.02, *p* = 0.03 in Figure 2D, respectively).

**TABLE 1:** Clinicopathological variables associated with FAR in discovery cohorts

Variables	FAR ≤ 0.0875 (*n* = 38)		FAR > 0.0875 (*n* = 78)		Total (*n* = 116)	*p* value
Age (year), n, %						0.113
<65	24	63.2%	36	46.2%	60	
≥65	14	36.8%	42	53.8%	56	
Gender, n, %						0.475
Male	19	50%	32	41.0%	51	
Female	19	50%	46	59.0%	65	
TNM stage, n, %						0.117
I‐II	33	86.8%	56	71.8%	89	
III‐IV	5	13.2%	22	28.2%	27	
Tumor differentiation, n, %						0.004
Low	5	13.2%	25	32.1%	30	
High	33	86.8%	53	67.9%	86	
Tumor size (cm), n, %						0.131
<5	22	57.9%	32	41.0%	54	
≥5	16	42.1%	46	59.0%	62	
Tumor number, n, %						0.057
Single	31	81.6%	73	93.6%	104	
Multiple	7	18.4%	5	6.4%	12	
HBsAg, n, %						0.475
Negative	21	55.3%	50	64.1%	71	
Positive	17	44.7%	28	35.9%	45	
Lymph node invasion, n, %						0.283
No	34	89.5%	62	79.5%	96	
Yes	4	10.5%	16	20.5%	20	
Vascular invasion, n, %						0.756
No	30	78.9%	58	74.4%	88	
Yes	8	21.1%	20	25.6%	28	
Perineural invasion, n, %						0.196
No	33	86.8%	58	74.4%	91	
Yes	5	13.2%	20	25.6%	25	
Smoking history, n, %						1
No	28	73.7%	57	73.1%	85	
Yes	10	26.3%	21	26.9%	31	
Drinking history, n, %						1
No	28	73.7%	59	75.6%	87	
Yes	10	26.3%	19	24.4%	29	
Liver cirrhosis, n, %						1
No	30	78.9%	61	78.2%	91	
Yes	8	21.1%	17	21.8%	25	
Portal hypertension, n, %						0.102
No	35	92.1%	77	98.7%	112	
Yes	3	7.9%	1	1.3%	4	
Child–Pugh grade, n, %						0.139
A	36	94.7%	65	83.3%	101	
B	2	5.3%	13	16.7%	15	
BMI (Kg/m^2^), n, %						0.265
≤22.66	17	44.7%	45	57.7	62	
>22.66	21	55.3%	33	42.3%	54	
AFP (µg/L), n, %						0.037
≤9	33	86.8%	76	97.4%	109	
>9	5	13.2%	2	2.6%	7	
CEA (µ/L), n, %						0.187
≤5	31	81.6%	53	67.9%	84	
>5	7	18.4%	25	32.1%	32	
CA19‐9 (µ/ml), n, %						0.002
≤37	22	57.9%	21	26.9%	43	
>37	16	42.1%	57	73.1%	73	
Serum albumin (g/L), n, %						<0.001
≤35	1	2.6%	25	32.1%	26	
>35	37	97.4%	53	67.9%	90	
Fibrinogen						0.032
≤4	1	2.6%	15	19.2%	16	
>4	37	97.4%	63	87.8%	100	
PMI, n, %						0.825
Low	15	39.5%	34	43.6%	49	
High	23	60.5%	44	56.4%	67	

Abbreviations: AFP, alpha‐fetoprotein; BMI, body mass index; CA19‐9, Carbohydrate antigen199; CEA, carcinoembryonic antigen; FAR, fibrinogen‐albumin ratio; PMI, psoas muscle index.

### 3.2 Prognosis of patients and baseline characteristics

For the discovery cohort, the average age of the patient was 64.94 ± 10.12 years, 51 (44%) patients were men and 65 (56%) were women. Most patients were classified as AJCC8th I and II stage (76.7%), there were 86 (74.1%) patients whose tumor differentiation is well‐differentiated. Tumor size in 54 (46.6%) patients was less than 5 cm and 104 (89.7%) patients had a single tumor. The median PMI for men was 8.86 cm^2^/m^2^ (IQR, 7.31–11.11) and 6.43 cm^2^/m^2^ (IQR, 5.35–8.39). Sex‐specific cut‐off values of PMI were calculated in the discovery cohort using X‐tile software, men's PMI cut‐off value was 8.6 cm^2^/m^2^, for women was 6.0 cm^2^/m^2^, we defined a PMI below 8.6 cm^2^/m^2^ in men and 6.0 cm^2^/m^2^ in women as sarcopenia. The baseline characteristics of the discovery and validation cohorts are presented in Table 2. The median follow‐up time was 33.9 months (1.05–79.64 months) and the median OS was 11.80 months, the OS probabilities at 1 year, 3 years, and 5 years were 66.4%, 30.0%, and 19.0%, respectively. The median RFS was 8.01 months, the RFS probabilities at 1 year, 3 years, and 5 years were 45.8%, 21.7%, and 16.7%, respectively. A total of 68 ICC patients were enrolled in the validation cohort, of those, the median follow‐up time was 20.7 months (2.04–52.51 months) and the median OS was 13.21 months, the estimated OS rate at 1 year and 3 years was 68.5% and 36.4%. The median RFS was 11.76 months, and the estimated RFS rate at 1 year and 3 years was 63.0% and 36.4%, respectively.

**TABLE 2:** Clinical and pathological characteristics of patients in the discovery and validation cohorts

Variables	Discovery cohort (*n* = 116)		Validation cohort (*n* = 68)		Total (*n* = 184)	*p* value
Age (year), *n*, %						0.695
<65	60	51.7%	38	55.9%	98	
≥65	56	48.3%	30	44.1%	86	
Gender, *n*, %						0.073
Male	51	44.0%	40	58.8%	91	
Female	65	56.0%	28	41.2%	93	
TNM stage, *n*, %						0.017
I‐II	89	76.7%	40	58.8%	129	
III‐IV	27	23.3%	28	41.2%	55	
Tumor differentiation, *n*, %						0.003
Low	30	25.9%	33	48.5%	63	
High	86	74.1%	35	51.5%	121	
Tumor size (cm), *n*, %						0.053
<5	54	46.6%	21	30.9%	75	
≥5	62	53.4%	47	69.1%	109	
Tumor number, *n*, %						1
Single	104	89.7%	61	89.7%	165	
Multiple	12	10.3%	7	10.3%	19	
HBsAg, *n*, %						0.009
Negative	71	61.2%	55	80.9%	126	
Positive	45	38.8%	13	19.1%	58	
Lymph node invasion, *n*, %						0.904
No	96	82.8%	55	80.9%	151	
Yes	20	17.2%	13	19.1%	33	
Vascular invasion, *n*, %						0.112
No	88	75.9%	59	86.8%	147	
Yes	28	24.1%	9	13.2%	37	
Perineural invasion, *n*, %						0.343
No	91	78.4%	58	85.3%	149	
Yes	25	21.6%	10	14.7%	35	
Smoking history, *n*, %						0.039
No	85	73.3%	39	57.4%	124	
Yes	31	26.7%	29	42.6%	60	
Drinking history, *n*, %						0.012
No	87	75%	38	55.9%	125	
Yes	29	25%	30	44.1%	59	
Liver cirrhosis, *n*, %						0.487
No	91	78.4%	57	83.8%	148	
Yes	25	21.6%	11	16.2%	36	
Portal hypertension, *n*, %						0.653
No	112	96.6%	67	98.5%	179	
Yes	4	3.4%	1	1.5%	5	
Child–Pugh grade, *n*, %						0.907
A	101	87.1%	58	85.3%	159	
B	15	12.9%	10	14.7%	25	
BMI (Kg/m^2^), *n*, %						0.207
≤22.66	62	53.4%	29	42.6%	91	
>22.66	54	46.6%	39	57.4%	93	
AFP (µg/L), *n*, %						0.275
≤9	109	94.0%	60	88.2%	169	
>9	7	6.0%	8	11.8%	15	
CEA (µ/L), *n*, %						0.758
≤5	84	72.4%	47	69.1%	131	
>5	32	27.6%	21	30.9%	53	
CA19‐9 (µ/ml), *n*, %						0.934
≤37	43	37.1%	24	35.3%	67	
>37	73	62.9%	44	64.7%	117	
Serum albumin (g/L), *n*, %						0.062
≤35	26	22.4%	7	10.3%	33	
>35	90	77.6%	61	89.7%	151	
FAR, *n*, %						<0.001
≤0.0875	38	32.8%	40	58.8%	78	
>0.0875	78	67.2%	28	41.2%	106	
PMI, *n*, %						0.169
Low	49	42.2%	21	30.9%	70	
High	67	57.8%	47	69.1%	114	

Abbreviations: AFP, alpha‐fetoprotein; BMI, body mass index; CA19‐9, Carbohydrate antigen199; CEA, carcinoembryonic antigen; FAR, fibrinogen‐albumin ratio; PMI: psoas muscle index.

### 3.3 Independent risk factors for OS and RFS in the discovery cohort

To determine the potential independent risk factors for OS and RFS in the discovery cohort, univariate and multivariate Cox proportional regression analyses were performed. As shown in Figure 2, age, TNM stage, tumor differentiation, BMI, CEA, CA19‐9, FAR, and PMI were risk factors related to both OS and RFS in univariate analysis. These eight factors were enrolled in the multivariate model, the result of multivariate analysis suggested that age (HR: 1.68, 95% CI: 1.00–2.80, *p* = 0.049), FAR (HR: 2.00, 95% CI: 1.04–3.85, *p* = 0.038), and PMI (HR: 0.35, 95% CI: 0.21–0.59, *p* < 0.001) were significant independent risk factors for OS. FAR (HR: 2.07, 95% CI: 1.07–4.02, *p* = 0.03) and PMI (HR: 0.41, 95% CI: 0.24–0.68, *p* < 0.001) were also significant independent risk factors for RFS in multivariate analysis.

### 3.4 The prognostic value of FAR combine with sarcopenia in ICC patients

**FIGURE 3:**
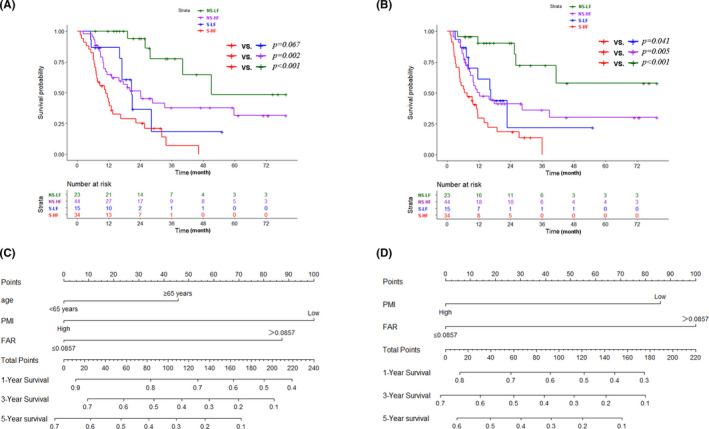
Kaplan‐Meier curves of OS (A) and RFS (B) stratified according to sarcopenia and FAR, and the nomogram of OS (C) and RFS (D) in discovery cohort. Abbreviations: FAR, fibrinogen‐albumin ratio; PMI, psoas muscle index; HF, high FAR; LF, low FAR; NS, non‐sarcopenia; S, sarcopenia; OS, overall survival; PMI, psoas muscle index; RFS, recurrence‐free survival

According to the level of FAR and sarcopenia, we divided the patients in the discovery cohort into four groups, sarcopenia with high FAR (S‐HF), sarcopenia with low FAR (S‐LF), non‐sarcopenia with high FAR (NS‐HF), and non‐sarcopenia with low FAR (NS‐LF). The survival curve showed the median OS of the S‐HF group was 10.8 months, S‐LF group was 20.7 months, NS‐HF group was 24.2 months, and NS‐LF group was 51.2 months. Furthermore, the survival of S‐HF and NS‐LF (*p *< 0.001), and S‐HF and NS‐HF (*p* = 0.002) had a significant difference, However, there was no significant difference between S‐HF and S‐LF (*p* = 0.067 Figure 3A). Moreover, the median RFS of group S‐HF, S‐LF, NS‐HF were 6.6 months, 16.6 months, and 12.3 months, respectively. Obvious differences were observed between S‐HF and NS‐LF (*p *< 0.001), S‐HF and NS‐HF (*p* = 0.005), and S‐HF and S‐LF (*p* = 0.041 Figure 3B).

### 3.5 Development and verification of the OS and RFS nomograms

**FIGURE 4:**
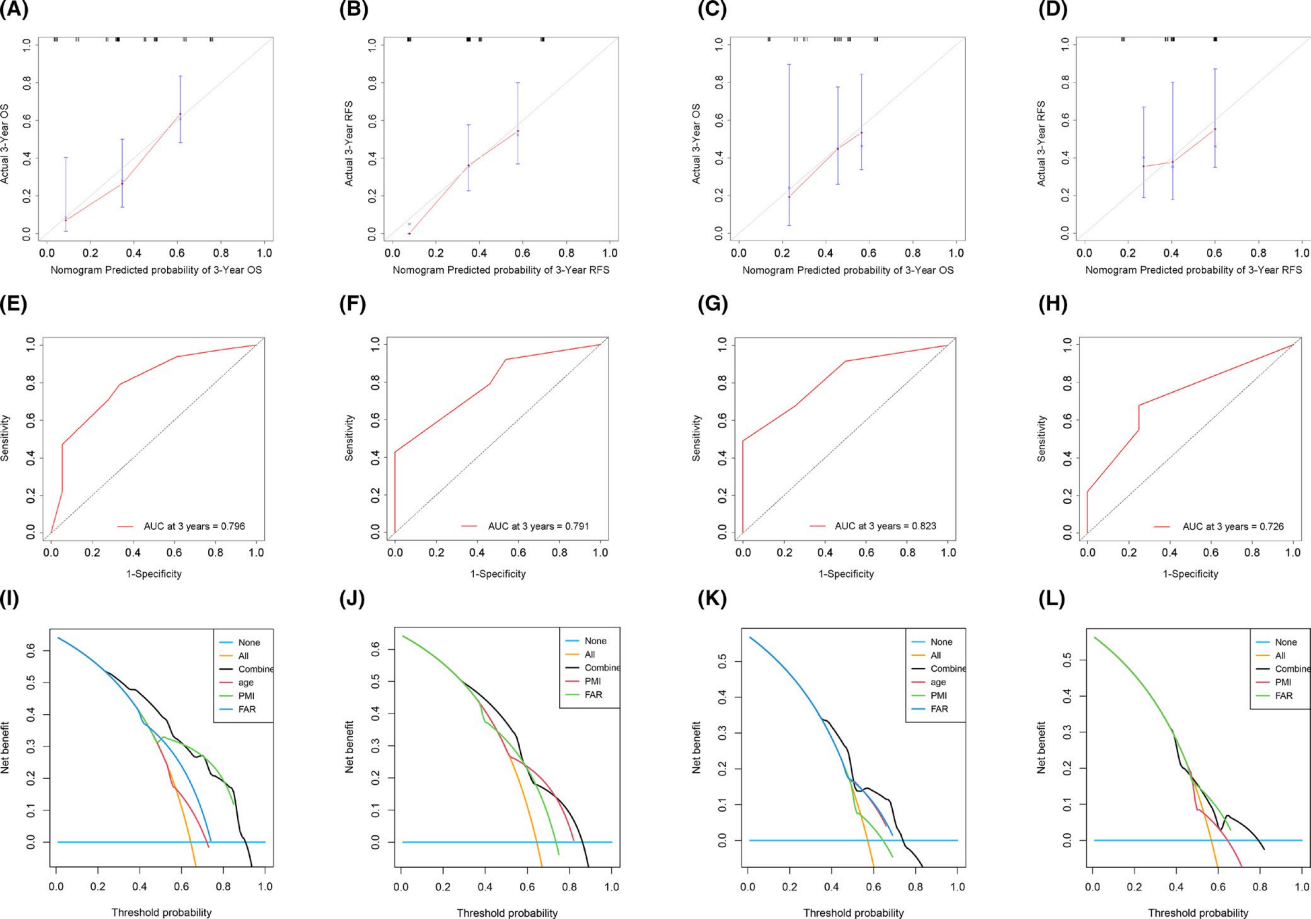
The internal and external verification of the predictive nomogram. (A) The calibration plot for predicting three‐year OS of discovery cohort, (B) RFS of discovery cohort, (C) three‐year OS of validation cohort, (D) and RFS of validation cohort, (E) ROC curve for predicting three‐year OS of discovery cohort, (F) RFS of discovery cohort, (G) three‐year OS of validation cohort, (H) and RFS of validation cohort, (I) DCA curve show prediction for three‐year OS in the discovery cohort, (J) RFS in discovery cohort, (K) three‐year OS of validation cohort, (L) and RFS of validation cohort. The black solid line represents the combined nomogram. Abbreviations: AUC, the area under ROC curve; DCA, Decision curve analysis; OS, overall survival; RFS, recurrence‐free survival; ROC, Receiver operating characteristic

**FIGURE 5:**
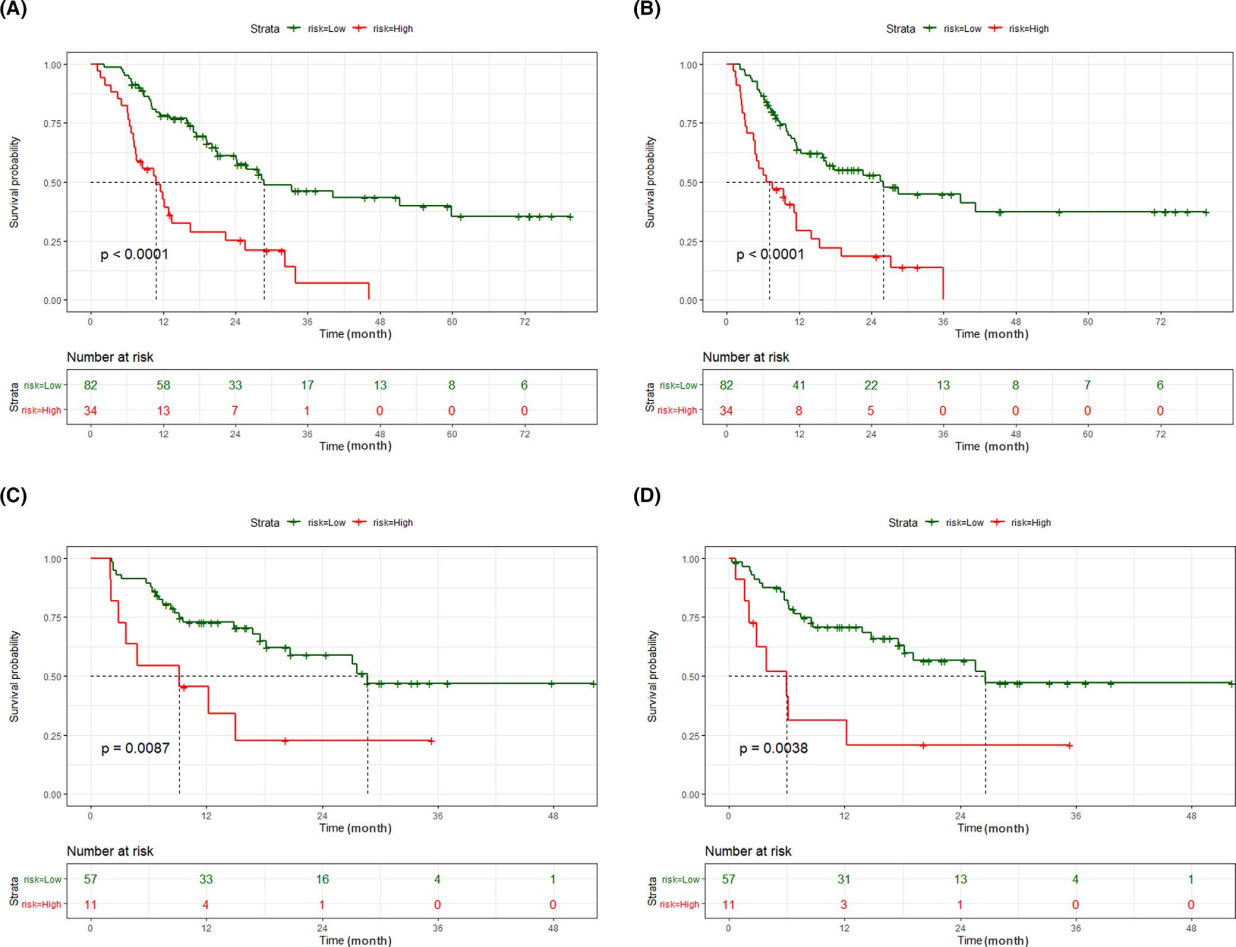
Kaplan‐Meier curves of risk stratification for ICC patients. The Kaplan‐Meier curves of OS (A) and RFS (B) for risk stratification according to nomogram score in discovery cohort, and OS (C) and RFS (D) in validation cohort. Abbreviations: ICC, intrahepatic cholangiocarcinoma; OS, overall survival; RFS, recurrence‐free survival

Based on the independent risk factors in the multivariate analysis, two nomograms were constructed to predict the OS and RFS of ICC patients (Figure 3C and 3D). The C‐index of two nomogram models reached 0.713 and 0.686 in the discovery cohort. The calibration curves showed good consistency between the nomogram prediction and actual survival for 3‐year OS and RFS in the discovery cohort (Figure 4A and 4B) and validation cohort (Figure 4C and 4D). In addition, the ROC curves demonstrated good discrimination to predict the 3‐year OS and RFS in the discovery cohort, the AUC was 0.796 and 0.791 (Figure 4E and 4F). Similarly, high AUC was also noticed for the validation cohort, the AUC of OS and RFS were 0.823 and 0.726, respectively (Figure 4G and 4H). The DCA curves revealed that compared with PMI and FAR, the combined model had better net benefits both in the discovery cohort (Figure 4I and 4J) and validation cohort (Figure 4K and 4L). In order to evaluate the discriminatory of these two nomograms, the patients in the discovery and the verification cohort were divided into the high‐risk group and low‐risk group based on the risk scores calculated by each nomogram. The K–M analysis revealed that patients in the high‐risk group had shorter OS and RFS than patients in the low‐risk group (Figure 5).

## 4 DISCUSSION

Increasing evidence showed that systemic inflammatory response^30^ and malnutrition^31^ were important factors affecting the prognosis of ICC patients after surgery. As a new indicator of inflammation, FAR was related to the prognosis of many malignant tumors, but its role in ICC was not yet clear. Sarcopenia usually represented malnutrition in patients because of the decline of skeletal muscle mass. This study demonstrated that the FAR was an independent risk factor for the prognosis of ICC patients after surgery and combined with sarcopenia to construct a prognostic nomogram model. After various methods of verification, the nomogram models we built showed satisfactory prediction performance.

Recently, Systemic inflammation was widely recognized to participate in tumorigenesis and cancer progression, by facilitating tumor angiogenesis, stimulating the proliferation of cancer cells, and accelerating tissue infiltration.^32^ Various inflammatory biomarkers can effectively represent the systemic inflammatory response, so they were often used to predict the prognosis of cancer patients. In past reports, a large number of inflammatory indicators had been reported to have close relation to the prognosis of patients with intrahepatic cholangiocarcinoma, including platelet to lymphocyte ratio (PLR), neutrophil to lymphocyte ratio (NLR), lymphocyte to monocyte ratio (LMR), C‐reactive protein (CRP), and Glasgow Prognostic Score (GPS).^33^, ^34^, ^35^ Recently, another inflammation biomarker FAR was found to be associated with the prognosis of manycancers.^19^, ^36^, ^37^ To our best knowledge, this was the first study to prove that FAR was of great significance in predicting the prognosis of ICC. Moreover, FAR would be a valuable biomarker for predicting the prognosis of ICC, because it can be easily obtained from peripheral blood examinations.

In our study, PMI is considered as a symbol to define sarcopenia, because it is relatively easy to obtain and studies have proven that it is feasible.^28^, ^38^ Thus far, no standard reference value for PMI has been determined. In our study, optimal cut‐off values for PMI were calculated by X‐tile software in the discovery cohort, the result showed that PMI cut‐off value in men was 8.6 cm^2^/m^2^, for women was 6.0 cm^2^/m^2^, which was different from the result of Hahn's study among the Germans (5.7 cm^2^/m^2^ in men, 5.1 cm^2^/m^2^ in women)^39^ and Chakedis's study among the Americans (7.9 cm^2^/m^2^ in men, 6.5 cm^2^/m^2^ in women).^40^ These differences may be caused by different stratification methods and different ethnic groups. Okumura et al. confirmed that sarcopenia had a closed relation to the survival of ICC patients with TNM stage I‐III. We also proved that sarcopenia was a potential prognostic factor in ICC patients after surgery, which was in line with previous reports.^39^, ^41^ In past studies, either FAR or sarcopenia was often used as a single predictor rather than a combined indicator. We are the first to combine FAR with sarcopenia to predict the prognosis of ICC patients underwent radical surgery, and the results show that patients with sarcopenia and high FAR have significantly shorter survival time than other patients. These results are consistent with our clinical experience, patients with preoperative high inflammation and preoperative low nutrition have poorer outcomes. In order to test the reliability and practicability of this conclusion, we established the nomograms for OS and RFS then carried out external verification, the results of external verification were satisfactory.

There are some limitations to this study. First, this study only included patients with ICC who underwent radical surgery. The nomograms we established may not be suitable for patients with ICC who received other treatments, such as palliative surgery and chemotherapy. Second, this study has internal verification and external verification, however, the patients included in our study are from China, future studies are needed to verify this conclusion in different races and regions. Third, in addition to the factors that associated with the prognosis of patients discussed in this study, there were many factors not included in this study, such as resection margin, involvement of extrahepatic margin, type of tumor (mass forming, periductal infiltrating type, intraductal growth type). In short, more studies are needed to further verify the clinical application of FAR and sarcopenia in patients with ICC in the future.

## 5 CONCLUSIONS

In conclusion, high levels of FAR and sarcopenia are related to the poor prognosis of ICC patients, which are independent risk factors for OS and RFS in ICC patients undergoing radical surgery. FAR and sarcopenia are convenient, inexpensive, and reliable marks that provide references for improving the prognosis and new treatment strategies for ICC patients after surgery.

## CONFLICT OF INTEREST

All authors declare no conflict of interest.

## ETHICS STATEMENT

This study was approved by the Medical Ethics Committee of the First Affiliated Hospital of Wenzhou Medical University and Qilu Hospital of Shandong University. All patients signed written informed consent.

## Data Availability

The data, models, and code generated or used during the study are available from the corresponding author by reasonable request.
